# Optoelectronic Properties of C_60_ and C_70_ Fullerene Derivatives: Designing and Evaluating Novel Candidates for Efficient P3HT Polymer Solar Cells

**DOI:** 10.3390/ma12142282

**Published:** 2019-07-16

**Authors:** Juganta K. Roy, Supratik Kar, Jerzy Leszczynski

**Affiliations:** Interdisciplinary Center for Nanotoxicity, Department of Chemistry, Physics and Atmospheric Sciences, Jackson State University, Jackson, MS 39217, USA

**Keywords:** DFT, fullerene derivative, P3HT, polymer solar cell, QSPR, TD-DFT

## Abstract

Ten novel fullerene-derivatives (FDs) of C_60_ and C_70_ had been designed as acceptor for polymer solar cell (PSC) by employing the quantitative structure-property relationship (QSPR) model, which was developed strategically with a reasonably big pool of experimental power conversion efficiency (PCE) data. The QSPR model was checked and validated with stringent parameter and reliability of predicted PCE values of all designed FDs. They were assessed by the applicability domain (AD) and process randomization test. The predicted PCE of FDs range from 7.96 to 23.01. The obtained encouraging results led us to the additional theoretical analysis of the energetics and UV-Vis spectra of isolated dyes employing Density functional theory (DFT) and Time-dependent-DFT (TD-DFT) calculations using PBE/6-31G(d,p) and CAM-B3LYP/6-311G(d,p) level calculations, respectively. The FD4 is the best C_60_-derivatives candidates for PSCs as it has the lowest exciton binding energy, up-shifted lowest unoccupied molecular orbital (LUMO) energy level to increase open-circuit voltage (V_OC_) and strong absorption in the UV region. In case of C_70_-derivatives, FD7 is potential candidate for future PSCs due to its strong absorption in UV-Vis region and lower exciton binding energy with higher V_OC_. Our optoelectronic results strongly support the developed QSPR model equation. Analyzing QSPR model and optoelectronic parameters, we concluded that the FD1, FD2, FD4, and FD10 are the most potential candidates for acceptor fragment of fullerene-based PSC. The outcomes of tactical molecular design followed by the investigation of optoelectronic features are suggested to be employed as a significant resource for the synthesis of FDs as an acceptor of PSCs.

## 1. Introduction

Polymer solar cell (PSC) is a subject of discussion over the last decade due to its initial encouraging power conversion efficiency (PCE). Over time, organic dye-sensitized solar cells (DSSCs) and perovskite solar systems outperform the PSCs based on better and efficient PCE. Additionally, high cost and low-life time factors are other threats, which pose a great task for the researchers [1,2,3,4]. The PSC functions similarly to another kind of solar cell through the conversion of photons into an electrical current. The most common types of PSC are fullerene-based and non-fullerene based where they act as acceptor fragment and the role of polymer is a donor. Cuiet al. [2] experimentally showed that chlorinated non-fullerene acceptor-based PSC converts 16.5% of solar energy into an electrical current which is the highest reported PCE value for any non-fullerene PSCs till date. On the contrary, Meng et al. [4] reported 17.3% conversion employing tandem cell strategy using PTB7-Th:O6T-4F:PC_71_BM (PTB7-Th is poly[4,8-bis(5-(2-ethylhexyl)thiophen-2-yl)benzo[1,2-b:4,5-b′] dithiophene-co-3-fluorothieno[3,4-b]thiophene-2-carboxylate]; O6T-4F is carbon-oxygen-bridged i8 difluoro-substituted 1,1-dicyanomethylene-3-indanone; PC_71_BM is [6,6]-phenyl C71 butyric methyl ester) with an architecture of ITO/ZnO/PFN-Br/active-layer/M-PEDOT/Ag and ITO/ZnO/active-layer/MoO3/Ag where PC71BM acts as acceptor material under fullerene-based PSC. Fullerene derivative (FD) PC_61_BM conjugated with diverse polymers P3HT (poly(3-hexyl)thiophene), PTPTB (poly-N-dodecyl-2,5,-bis(2’-thienyl)pyrrole, 2,1,3-benzothiadiazole), PEOPT (poly(3-(4’-(1′′,4′′,7′′-trioxaoctyl)phenyl)thiophene), PFDTBT (poly{[2,7-(9-(20-ethylhexyl)-9-hexylfluorene])-alt-[5,50-(40,70-di-2-thienyl-20,10,30-benzothiadiazole)]}) showed PCE values range from 2.8 to 4.4 [5,6,7,8], 1.7–2.1 [9], 1.75 [9] 1.9 [9], respectively. Even, PCPDTBT (poly[2,6-(4,4-bis(2-ethylhexyl)-4H-cyclopenta[2,1-b;3,4-b′]dithiophene)-alt-4,7(2,1,3-benzothiadiazole)]) and PBDTP-DTBT (poly{4,8-bis(4-(2-ethylhexyl)-phenyl)- benzo[1,2-b:4,5-b′]dithiophene-alt-[4,7-di(4-(2-ethylhexyl)-2- thienyl)-2,1,3-benzothiadiazole)-5, 5′-diyl]}) showed PCE of 3.2 [10] and 8.07 [11] with PC71BM, respectively. While, PCDTBT (poly[N-9′-heptadecanyl-2,7-carbazole-alt-5,5-(4′,7′-di-2-thienyl-2′,1′,3′-benzothiadiazole)]) and PCPDTBT offers PCE of 6.1 [12] and 6.16 [13] with PC_70_BM, respectively. 

The PCE value of existing PSCs is reasonable but not better than other commercial solar cell systems. Thus, improved and efficient PSCs are required by the implementation of rational designing of different fragments followed by optoelectronic properties evaluation to establish them as a future system. Based on the above discussion, it is quite clear that a good amount of simple and complex polymers has been examined, but the PCE value has not improved drastically. Therefore, we have aimed for novel modifications of FDs to improve the PCE of PSCs. Arbitrarily synthesizing various FDs is not a practical solution, as well as it is costly and time-consuming. Thus, considering experimental PCE data of existing FDs, in silico models can be prepared by quantitative structure-property relationship (QSPR) model [14]. Our group has proposed the first QSPR model followed by the virtual screening of FDs generating future lead acceptor fragment for PSC with PCE value of 12.11% [15]. Additionally, the QSPR technique was successfully employed in all steps from designing to prediction purpose for DSSCs by our group [16,17] and other researchers [18,19] with encouraging outcomes. Therefore, without any doubt, the QSPR modeling can be tactfully employed for designing better acceptor FDs for PSCs.

In continuation of our previous work [15], in the present study, we have prepared a QSPR model followed by implementation of the mechanistic interpretation and identified vital structural fragments obtained from the model to design new FDs as an acceptor for PSC. Previously we had used the QSPR model to screen 169 FDs to find the best FDs based on PCE value only without considering identified features from the model [15]. In the present study, designing will help us to consider the structural fragments more precisely and effectively. Ten FDs have been designed including seven C_60_ and three C_70_ FDs. The PCE of designed FDs are predicted employing the developed QSPR model prepared from 59 existing experimental PCE data of FDs. Top four lead acceptors (3 from C_60_ and 1 C_70_) are further employed for energetics study along with analysis of UV-Vis spectra of isolated dyes. The rational scheme from designing to the electrochemical analysis of FDs for PSCs offers a detailed idea of how one can implement a QSPR model to design various future solar cells, not confined to only PSCs pool of species. 

## 2. Materials and Methods 

### 2.1. QSPR Modeling Study and Designing

#### 2.1.1. Dataset 

A series of 59 FDs consist of 52 C_60_ and 7 C_70_ derivatives as acceptor for PSCs with experimental PCE data were taken from our previous research [15] to generate a statistically acceptable and predictive QSPR model. The experimental data for all solar cells is measured according to bulk-heterojunction (BHJ) devices, where FD acts as the electron acceptor and Poly(3-hexylthiophene) (P3HT), a commonly used photovoltaic polymer as the donor material. The experimental data and chemical structures of FDs have been reported in Table S1 in the Supplementary material section.

#### 2.1.2. Descriptors Calculation

Molecular structures of FDs had been drawn in GaussView 6.0 [20] and optimized by semi-empirical PM6 method using Gaussian 16 software [21]. The output structures were employed in MarvinView (ChemAxon) [22] software to calculate physicochemical and solvent accessible surface area descriptors. To compute Simplex Representation of Molecular Structure (SiRMS) [23,24] descriptors, QSAR4U software was applied which helps to identify and understand the major structural fragments responsible for higher PCE. 

#### 2.1.3. Dataset Division and Modeling Tools

The entire pool of descriptors was treated with a 0.0001 variance cutoff and passed through a 0.99 correlation coefficient to eradicate the highly correlated feature and decrease the noise level among descriptors. Followed by the dataset is divided into 3:1 ratio randomly by generating training and test sets with 44 and 15 FDs, respectively. The training set was then employed to develop a PLS based model using Partial Least Squares version 1.0 tool [25].

#### 2.1.4. Model Validation and Designing Criteria

To assess the quality of a QSPR model followed by its prediction capability towards new compounds depend largely on statistical metrics. Internal metrics like R^2^ (goodness-of-fit) and leave-one-out cross-validation (Q^2^_LOO_) are important parameters. While external validation metric R^2^_pred_ or Q^2^_ext(F1)_ signify the predictability. Along with these classical parameters, to check the quality of the developed model, we have further employed stringent metrics like the r_m_^2^ metrics [26], the Q^2^_ext(F2)_ [27] and Golbraikh and Tropsha’s [28] criteria. To follow the Organization for Economic Co-operation and Development (OECD) principle 3, we have studied the applicability domain test employing the Euclidean distance approach [29]. To check the robustness of the model, Y-randomization technique had been performed to generate 100 random models [30]. The average R^2^ and Q^2^_LOO_ values of all 100 random models should be failed the stipulated threshold value of 0.5. 

### 2.2. Quantum Study of Designed FDs

To model P3HT polymer, an oligomer with 8 monomers considered to study the compatibility of the designed four FDs [31,32]. In all of the computations, PCBM was used as a reference. A variety of functionals [B3LYP, CAM-B3LYP, PBE] and basis sets [6-31G(d,p), 6-311G(d,p)] were used for the accurate description of the frontier orbitals of [6,6]-phenyl-C61-butyricacidmethylester (PCBM) and Poly(3-hexylthiophene) (P3HT) (see Table 1). PBE/6-31G(d,p) and CAM-B3LYP/6-311G(d,p) level of theory employed for the DFT and TDFT calculations, respectively. Absorption spectra of FDs in chlorobenzene solvent have been simulated by the Conductor like polarizable continuum model (C-PCM) [33] considering 20 low-lying singlet-singlet allowed transitions. All calculations were performed with the Gaussian 16 program package [21].

## 3. Results and Discussion

### 3.1. QSPR Modeling 

The training set offered 7 features (descriptors) and 6 latent variables (LVs) based PLS equation (Equation (1)). To judge the goodness-of-quality of the presented equation along with predictivity of the test set molecules, we have checked a series of stringent statistical metrics and all of them passed the stipulated threshold values (Table 2).

PCE (%) = 2.50 + 1.84*S_A(chg)/A_D_D_D/1_2s,1_3s,3_4a/6 −0.78*Fr5(chg)/B_C_C_C_D/1_4s,2_3s,2_4s,3_4s/ −0.06*Fr5(type)/C.3_C.3_C.3_C.3_H/1_2s,2_3s,3_4s,4_5s/ −0.937*Fr5(att)/C_C_E_E_E/1_3s,2_4s,3_5a,4_5a/ −0.11*Fr5(type)/C.3_C.3_C.AR_C.AR_C.AR/1_4s,2_3s,2_5s,4_5a/ +0.61*S_A(type)/C.3_C.3_C.3_C.AR/1_3s,2_3s,3_4s/5-0.008*ASA_P(1)

Considering the complexity and diversity of FDs structures, the internal and external prediction variances are 0.74 and 0.73, respectively which are highly acceptable in QSPR modeling. The same value for Q^2^_F1_ and Q^2^_F2_ suggested stability between training and test sets followed by identical distribution of FDs. The model also passed the strict r_m_^2^ metrics and Golbraikh and Tropsha’s criteria. To check the randomness of the model which one support that the model is not developed by chance, we have performed process validation by generation of 100 random models. We found that average R^2^ and Q^2^ values for 100 random models are 0.17 and −0.38, respectively which failed the stipulated value of 0.5 for both metrics. It supports the conclusion that the PLS model is not a result of correlation-by-chance. To check the applicability domain (AD), we have prepared ED-based AD study and found that all test compounds are falling within the AD zone created by the training set data which supports the reliability of prediction for test compounds. 

### 3.2. Mechanistic Interpretation of Model

Out of seven features, two features namely S_A(chg)/A_D_D_D/1_2s,1_3s,3_4a/6 and S_A(type)/C.3_C.3_C.3_C.AR/1_3s,2_3s,3_4s/5 contributed positively to PCE value. This signifies that the higher value of these features may increase the PCE value. On the contrary, the remaining five features affect the equation negatively suggesting lowering or no effect on the PCE value (absent of these features or fragments in the FDs). 

**Figure 1:**
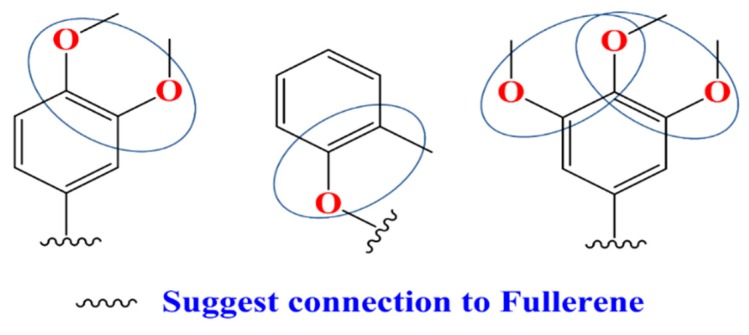
Fragments like -ortho directing groups substituted in the benzene ring help in power conversion efficiency (PCE) increment.

**Figure 2:**
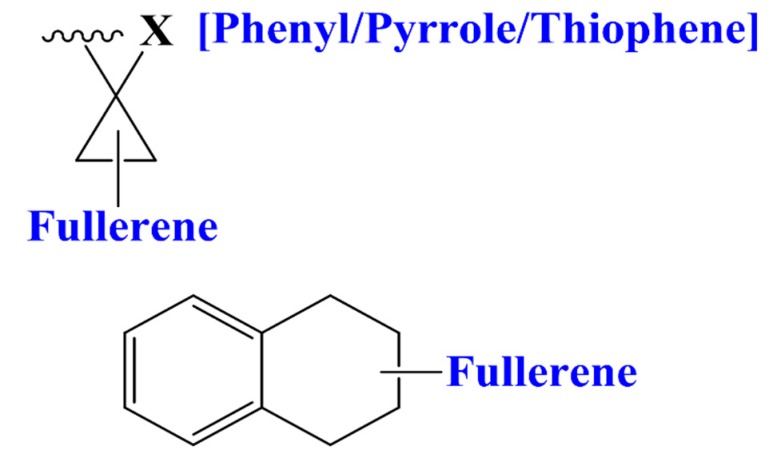
Important fullerene substituents for higher PCE.

**Figure 3:**
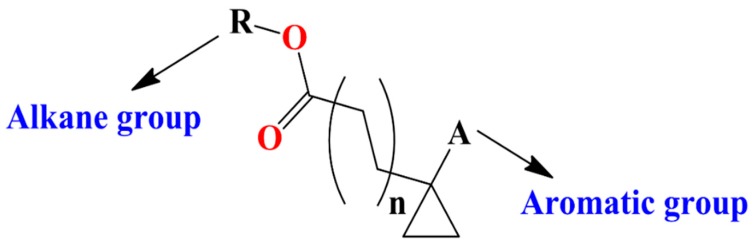
Fragment has detrimental effect on PCE value.

S_A(chg)/A_D_D_D/1_2s,1_3s,3_4a/6 defines a four-atomic fragment labeled by partial charges which are induced by -ortho directing groups in the benzene rings. FDs having the mentioned fragments (see Figure 1) have higher value for this descriptor and in a consequence promote higher PCE value. While, S_A(type)/C.3_C.3_C.3_C.AR/1_3s,2_3s,3_4s/5 represents types of fullerene substituent connections demonstrated in Figure 2 are also good for increment of PCE value. This specific fragment portrayed that aromatic rings like phenyl, thiophene, pyrrole (electron acceptors) attached to the fullerene by a linker help the electron withdrawing capability of the fullerene. Fr5(chg)/B_C_C_C_D/1_4s,2_3s,2_4s,3_4s/ also defines partial charges portrayed by the molecular fragment in Figure 3 offers detrimental effects to PCE. This suggests that it is better to avoid such specific substituents to FDs. Fr5(type)/C.3_C.3_C.3_C.3_H/1_2s,2_3s,3_4s,4_5s/ describes the existence of saturated carbon chains like [C(sp^3^)- C(sp^3^)- C(sp^3^)- C(sp^3^)-H]) and offers inductive effects and reduces the mesomeric effects of aromatic rings in a FD, which has a negative effect on PCE. Fr5(att)/C_C_E_E_E/1_3s,2_4s,3_5a,4_5a/ is associated to the van der Waals attraction between 3 or higher-ortho substituents in benzene rings with negative contribution to the PCE. Fr5(type)/C.3_C.3_C.AR_C.AR_C.AR/1_4s,2_3s,2_5s,4_5a/ portrayed substituents to a pentagon of the fullerene core which is electrochemically more steady than general two-points substituted FDs. Higher number of attachments in the parent fullerene affects the unsaturation and aromaticity negatively and results in reduction of acceptor property of FDs. ASA_P defines the solvent accessible surface area of polar atoms which is significant for the calculation of free energy changes as a result of shifting the molecule from a polar to a non-polar solvent during the formation of PSCs with BHJ layers. 

### 3.3. Designing of Novel FDs as Acceptor

**Figure 4:**
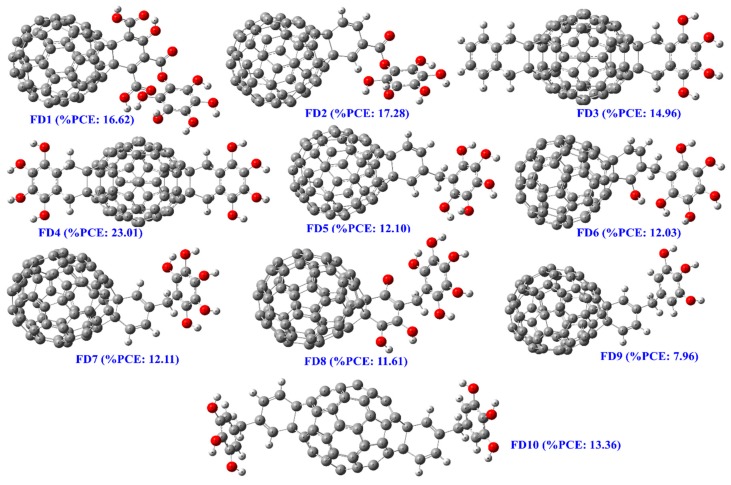
Structure of designed fullerene-derivatives (FDs) as lead acceptor molecule for polymer solar cells (PSCs).

Mechanistic interpretation of important fragments lead to higher PCE are considered here for the designing of lead FDs as acceptor for Fullerene-based PSCs. The major fragments illustrated in Figure 1 and Figure 2 are included in the acceptor and fragments (Figure 3, C(sp^3^)- C(sp^3^)- C(sp^3^)- C(sp^3^)-H, 3 or higher-ortho substituents in benzene and substituents to a pentagon of the fullerene core) which are detrimental to PCE are avoided when possible. Ten FDs structures (7 C_60_ and 3 C_70_) are designed (Figure 4) and modeled descriptors are calculated following similar protocols mentioned in Section 2.1.2. Followed by QSPR model (Equation (1)) is implemented to predict the PCE of the designed FDs and AD study has also been performed to check their prediction reliability. All 10 FDs passed the Euclidean distance-based AD study portraying the PCE values to be reliable and can be considered for the further introspection to prove them as future efficient acceptor for fullerene-based PSCs. The predicted PCE of FDs range from 7.96 to 23.01 considering both C_60_ and C_70_ FDs. Here, FD7, FD8 and FD9 are C_70_ FDs whose PCE values varies from 7.96 to 12.11, while remaining FDs are C60 FDs whose PCE values varies from 12.03 to 23.01. All values are no doubt encouraging and higher than any existing FD acceptor for PSCs. To check the electrochemical properties of these FDs, we have selected the top three C_60_ and top C_70_ FDs for further analysis. Computed modeled descriptors value along with mean normalized distance for AD study provided in Table S2 in the Supplementary material section.

### 3.4. Energetics of Isolated FDs

**Figure 5:**
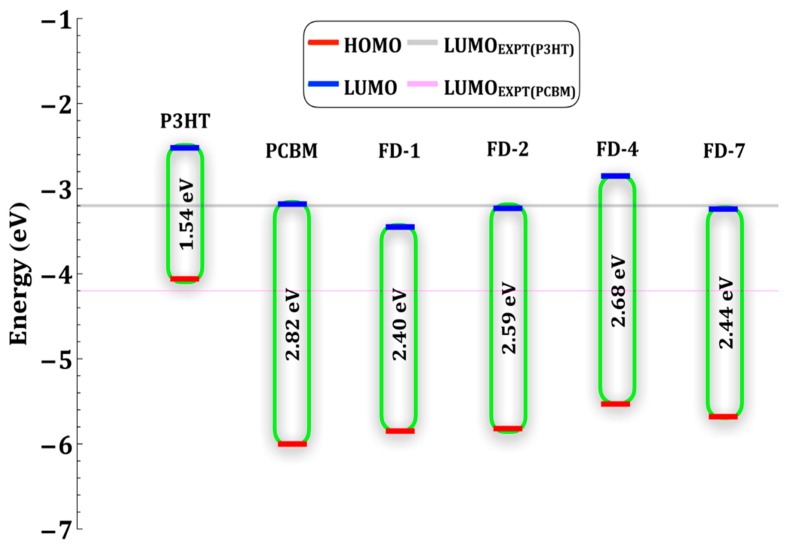
Computed energy diagram of the four FDs along with [6,6]-phenyl-C61-butyricacidmethylester (PCBM) and P3HT. All the values obtained with the use of PBE/6-31G(d,p) level of theory in the gas phase.

The computed HOMO energy of PCBM and P3HT are similar to experimental value while the LUMO values are overestimated by the chosen method which is accepted by the community [35]. According to McCormik et al. [35], B3LYP functional is sufficient to approximate HOMO energy of conjugated polymers while LUMO is not well approximated. Due to the overestimation of LUMO energy, the computed energy for HOMO-LUMO gap (E_gap_) is very high with B3LYP functional. To tradeoff between LUMO energy and E_gap_, we choose PBE functional for the ground state calculation. The computed value of the HL_g_ for the gas phase isolated PCBM is 2.82 eV but González et al. reported 1.48 eV (PBE-D3/TZP) [31] and by Thompson et al. it amounts to 1.4 eV [36] whereas Cook et al. reported experimental value for pure PCBM films to be 1.8 eV [34]. The computed energy gap for the isolated P3HT-8mer is 1.54 eV which is in good agreement with the reported results 1.31 eV [31] and 1.49 eV [37], whereas the experimental value for pure P3HT films is 1.9 eV [34]. Computed energetics of P3HT, PCBM and four FDs are compiled in Figure 5. 

We also compute the different driving forces for the exciton binding, dissociation, charge-transfer, and open-circuit voltage (V_OC_) for FDs which affect the smooth flow of exciton in the donor/acceptor blends. The following definition used to compute driving forces in terms of energy [38]:

(2) $$\Delta E_{1}=E_{Donor}^{LUMO}-E_{Acceptor}^{LUMO}$$

(3) $$\Delta E_{2}=E_{Acceptor}^{LUMO}-E_{Donor}^{HOMO}=V_{OC}$$

(4) $$\Delta E_{3}=E_{Donor}^{HOMO}-E_{Acceptor}^{HOMO}$$

The difference between the LUMO levels of donor and acceptor, ∆E_1_, is responsible for the charge dissociation of the excitons in polymer donor to overcome the excitations binding energy. The typical exciton binding energy is ca. 0.3–0.5 eV, if it is too large then the exciton charge separation will require more energy and lowers V_OC_. The value of ∆E_2_ determines the V_OC_ which can be increased by up-shifted LUMO energy level of acceptors and thus higher efficiency of PSCs. ∆E_3_, the difference between the HOMO levels of donor and acceptor, affects the dissociation of electron-hole pairs in the donor/acceptor interface. If the HOMO levels of acceptor are too high, ∆E_3_ will be too small which hinders the dissociation of electron-hole pairs at interface in some extent [38]. So, it is necessary to maintain effective ∆E_3_ to maintain a smooth dissociation. But ∆E_3_ is not the sole factor which affects the efficiency of PSCs. Balancing between all the related factors (Table 3), FD4 will be efficient acceptor as it has the lower exciton binding energy and higher V_OC_ with least ∆E_3_ value.

### 3.5. UV-Vis Absorption Spectra of Isolated FDs

**Figure 6:**
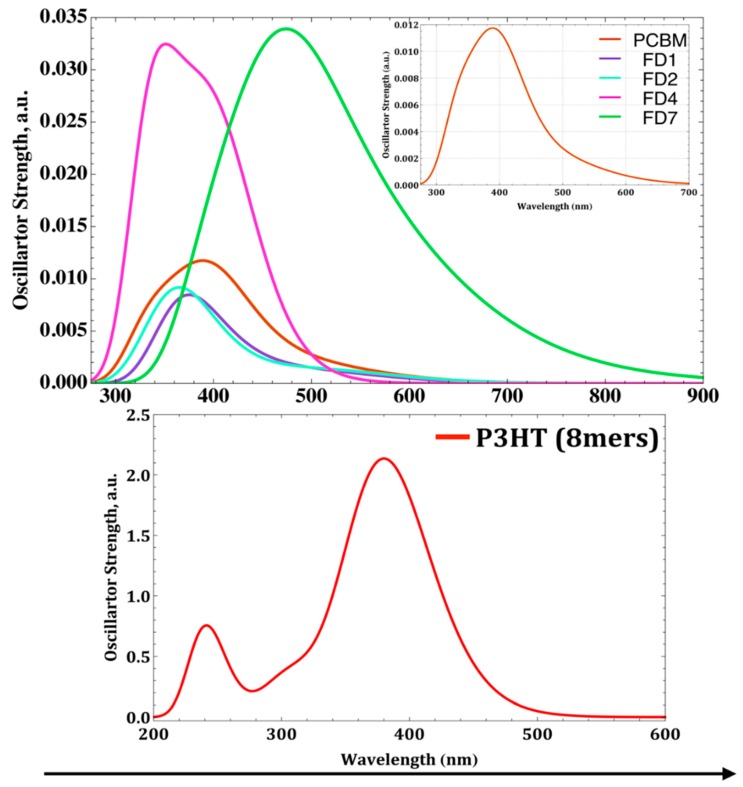
Simulated absorption spectra of PCBM, four FDs [Top] and P3HT [Bottom] with the use of TD/CAM-B3LYP/6-311G(d,p) level of theory in chlorobenzene solvent. Inset Top: Magnified PCBM absorption spectrum.

The Figure 6 shows the simulated absorption spectrum of the different PCBM, four designed FDs and P3HT. The spectrum of PCBM reproduces the qualitatively main features of reported experimental results [34,39], such as, it shows a strong optical absorption predominantly in the UV region, with very weak absorption (f = 0.0021) in the visible region (from 450 nm to 700 nm, see the inset in Figure 6). However, the spectrum of P3HT oligomer (Figure 6, Bottom) showing two strong absorption peaks at 241.20 nm (f = 0.3423) and 381 nm (f = 2.4886) with one shoulder at 307 nm (f = 0.4027), which represents a HOMO − LUMO + 6, HOMO − LUMO and HOMO − 1 − LUMO + 1 transition, respectively. As a reference, for pure P3HT films two maxima absorption peaks and one shoulder at 493 nm, 517 nm, and 572 nm, respectively have experimentally been reported, also attributed to the π-π* transitions [40,41,42]. Our simulated absorption of P3HT oligomer shows a small blue shift compare to experimental one.

Also, PCBM has weak absorption in visible region which is one of the factors that can be tuned to get better efficiency from PSCs [43]. If one examines the absorption strength of FDs, it is obvious that except FD4 and FD7 all of them having very weak absorption in the visible region. C_70_ derivatives FD7 is showing a red shift extends up to 600 nm with large oscillator strength [43]. From this aspect our designed FD4 and FD7 will be the most efficient acceptor in conjunction with donor P3HT.

## 4. Conclusions

In silico modeling followed by designing and optoelectronic properties evaluation of future lead FDs as acceptor for PSCs had been performed. The QSPR model led us to development of 10 novel FDs as acceptor including seven C_60_ and three C_70_. Based on the predicted PCE, optoelectronic properties of four FDs were evaluated by DFT and TDDFT. PBE/6-31G(d,p) and CAM-B3LYP/6-311G(d,p) level of theory were employed for gas phase DFT and solvent phase TDDFT computations. Frontier orbital energies and UV-Vis absorption spectra of the isolated P3HT oligomer, PCBM and FDs were analyzed to estimate the optoelectronic properties of four FDs as acceptor in future PSCs. Exciton binding energy plays the pivot role at interface when excitons diffuse and dissociate in to electrons on LUMO level of the acceptor. The big off-set of LUMO energy levels will hinders this process. FD4 is the best C_60_-derivatives candidates for PSCs as it has the lowest exciton binding energy, up-shifted LUMO energy level that assist to increase V_OC_ and strong absorption in the UV region. In case of C_70_-derivatives, FD7 is a potential candidate for future PSCs due to its strong absorption in UV-Vis region and lower exciton binding energy with higher V_OC_. By trading off the computed optoelectronic properties, our analysis supports our QSPR model which predict highest PCE values for FD4. 

The rational molecular modeling, designing, and prediction followed by quantum study offers valued reasoning for the synthesis of lead FDs with higher power conversion efficiency. The structural analysis concluded the following points:

- Ortho directing groups in the benzene rings and aromatic rings like phenyl, thiophene, pyrrole attached to the fullerene are significant features for better PCE of PSCs.
- Saturated carbon chains, 3 or higher -*ortho* substituents in benzene rings and a higher number of attachments in the parent fullerene core need to be avoided for higher PCE along with structural fragments with a lower solvent accessible surface area of polar atoms.

## Figures and Tables

**Table 1:** Energy profiles highest occupied molecular orbital (HOMO), lowest unoccupied molecular orbital (LUMO) and their gap (E_gap_) of the isolated P3HT oligomer, and PCBM. All the energies are in eV and PCBM/P3HT.

Method.	E_HOMO_	E_LUMO_	E_GAP_
PBE/6-31G(d,p)	−5.94/−4.00	−3.12/−2.46	2.82/1.54
PBE/6-311G(d,p)	−6.24/−4.49	−3.45/−2.43	2.79/2.06
B3LYP/6-31G(d,p)	−5.63/−4.76	−3.06/−1.81	2.57/2.95
B3LYP/6-311G(d,p)	−6.02/−5.08	−3.47/−1.92	2.55/3.16
CAMB3LYP/6-31G(d,p)	−6.78/−6.16	−2.08/−0.53	4.70/5.63
CAMB3LYP/6-311G(d,p)	−7.17/−6.36	−2.51/−0.77	4.66/5.59
Experiment [34]	6.0/5.2	4.2/3.2	1.8/2.0

**Table 2:** Obtained statistical data from the developed quantitative structure-property relationship (QSPR) model.

Validation	Metrics	Value	Threshold
Internal	N_Training_	44	-
	R^2^	0.74	>0.5
	Q^2^_LOO_	0.65	>0.5
	$\;\bar{r_{m(\mathrm{LOO})\mathrm{Scaled}}^{2}}$	0.54	>0.5
	${\Delta r}_{m(\mathrm{LOO})\mathrm{Scaled}}^{2}$	0.13	<0.2
External	N_Test_	15	-
	$Q_{F1}^{2}\;or\;R_{\mathrm{pred}}^{2}$	0.73	>0.5
	$Q_{F2}^{2}$	0.73	>0.5
	$\;\bar{r_{m(\mathrm{test})\mathrm{Scaled}}^{2}}$	0.64	>0.5
	${\Delta r}_{m(\mathrm{test})\mathrm{Scaled}}^{2}$	0.12	<0.2
Golbraikh and Tropsha’s criteria	r^2^	0.73	>0.5
	$\left|r_{0}^{2}-{r_{0}^{\prime }}^{2}\right|$	0.05	<0.3
	$\frac{r^{2}-r_{0}^{2}}{r^{2}}$	0.002	Any of them must be < 0.1
	$\frac{r^{2}-r^{\prime }{}_{0}^{2}}{r^{2}}$	0.06	
	k	1.01	$0.85\leq k\;\mathrm{or}\;k^{\prime }\;\leq 1.15\;$
	k’	0.91	

**Table 3:** Electronic energy level differences of P3HT and fullerene-derivatives (FDs) including [6,6]-phenyl-C61-butyricacidmethylester (PCBM).

FDs	$\Delta E_{1}$	$\Delta E_{2}$	$\Delta E_{3}$
PCBM	0.66	0.88	1.94
FD1	0.93	0.61	1.79
FD2	0.71	0.83	1.76
FD4	0.33	1.21	1.47
FD7	0.72	0.82	1.62

## Supplementary Materials

The following are available online at https://www.mdpi.com/1996-1944/12/14/2282/s1, Table S1: Fullerene derivatives with their experimental and predicted % PCE, Table S2: Computed value of modeled descriptors for each FDs along with their predicted % PCE and mean normalized distance value.
