# Supplementary material for: Optoelectronic Properties of C60 and C70 Fullerene Derivatives: Designing and Evaluating Novel Candidates for Efficient P3HT Polymer Solar Cells
Source: Materials (Basel). 2019 Jul 16;12(14):2282. doi: 10.3390/ma12142282 (PMC6678454; doi:10.3390/ma12142282)
Supplement: Supplementary file 1 [file materials-12-02282-s001.pdf]

materials

MDPI

Supplementary Information

Optoelectronic Properties of C60 and C70 Fullerene Derivatives: Designing and Evaluating Novel Candidates for Efficient P3HT Polymer Solar Cells

Juganta K. Roy †, Supratik Kar *,† and Jerzy Leszczynski *

Interdisciplinary Center for Nanotoxicity, Department of Chemistry, Physics and Atmospheric Sciences, Jackson State University, Jackson, MS 39217, USA

* Correspondence: supratik.kar@icnanotox.org (S.K.); jerzy@icnanotox.org (J.L.); Tel.: +1-601-979-0253 (S.K.); Fax: +1-601-979-7823 (J.L.)

† These authors contributed equally to this work.

Received: 24 June 2019; Accepted: 12 July 2019; Published: 16 July 2019

materials MDPI

Table S1. Fullerene derivatives with their experimental and predicted % PCE.

<table>
<tr><th rowspan="2">ID</th><th colspan="6">Substituents</th><th colspan="2">PCE (%)</th></tr>
<tr><th>R1</th><th>R2</th><th>R3</th><th>R4</th><th>R5</th><th>R6</th><th>Experimental</th><th>Predicted#</th></tr>
<tr><td colspan="9">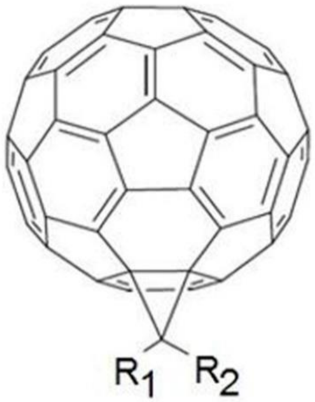
</td></tr>
<tr><td>1*</td><td>C_4H_9</td><td>C_6H_5</td><td>-</td><td>-</td><td>-</td><td>-</td><td>1.9</td><td>1.92</td></tr>
<tr><td colspan="9"></td></tr>
</table>

* Compounds present in the test set; #Predicted data employing PLS equation.

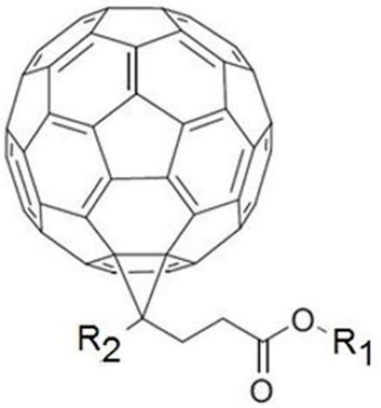

2	CH_3	C_6H_5	-	-	-	-	0.4	1.51
3	C3H7	C_6H_5	-	-	-	-	2.2	1.59
4	iso-Propyl	C_6H_5	-	-	-	-	2.8	1.64
5	C_4H_9	C_6H_5	-	-	-	-	2.7	1.29

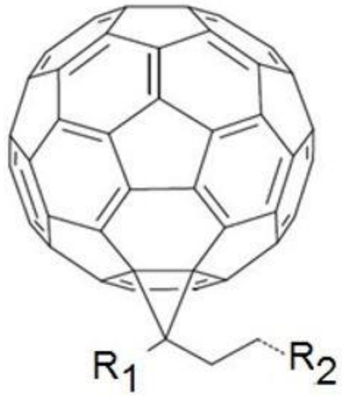

6*	-COOCH$_2$Ph	C$_6$H$_5$	-	-	-	-	2.5	1.66
7	-CH$_2$COOCH$_2$CH$_3$	C$_6$H$_5$	-	-	-	-	2.7	2.98
8	-CH$_2$COOCH$_3$	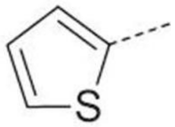	-	-	-	-	3.7	2.94
9	-COOCH$_3$	4-OCH3Ph	-	-	-	-	0.05	1.33
10	-COOC$_2$H$_5$	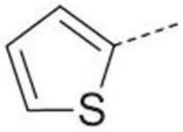	-	-	-	-	2.5	2.65
11*	-COOC$_3$H$_7$	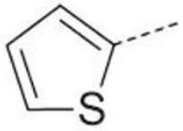	-	-	-	-	3.4	2.34
12	C$_4$H$_9$	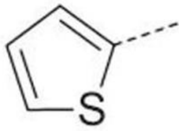	-	-	-	-	2.9	1.95

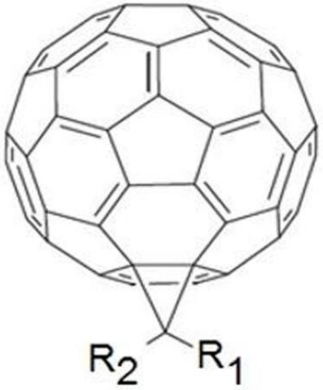								
13	C_6H_{13}	C_6H_5	-	-	-	-	2.8	2.58
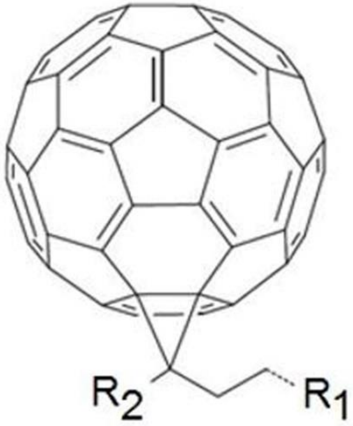								

14	C_4H_9	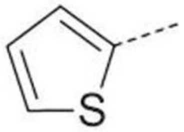	-	-	-	-	2.1	2.51
15	$-COOCH_3$	$(CH_2)_2COOCH_3$	-	-	-	-	0.02	0.76
16*	$-CH_2CH_2COOCH_2CH_2OCH_3$	$-COOC_2H_5$	-	-	-	-	0.9	0.87
17	H	COOC8H17	-	-	-	-	0.3	0.65
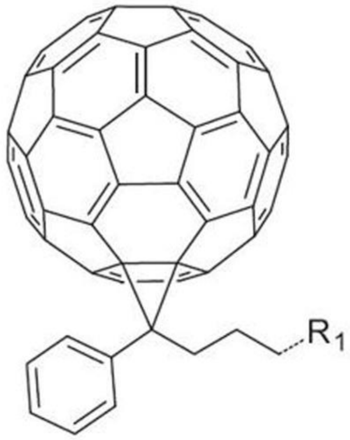								
18	$-COOCH_3$	-	-	-	-	-	3.5	2.91
19*	CH_2COOCH_3	-	-	-	-	-	2.3	2.75
20	$(CH_2)_2COOCH_3$	-	-	-	-	-	3.6	2.39
21	$(CH_2)_3COOCH_3$	-	-	-	-	-	2.8	2.18

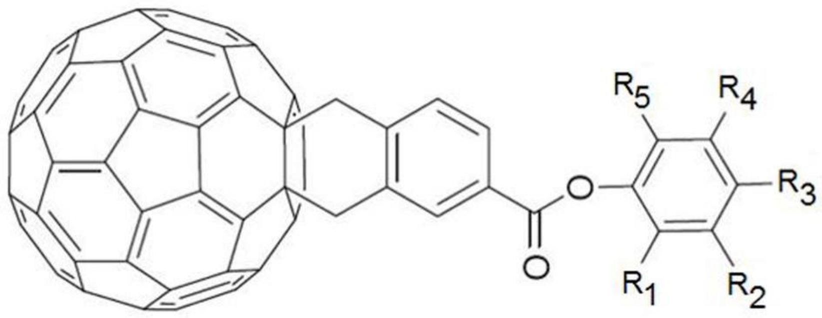

22	H	H	H	H	H	-	4.2	4.05
23*	H	H	OCH_3	H	H	-	3.6	4.10
24	H	OCH_3	OCH_3	OCH_3	H	-	1.2	1.00
25	H	H	F	H	H	-	3.5	3.72
26	F	F	F	F	F	-	0.6	-0.02

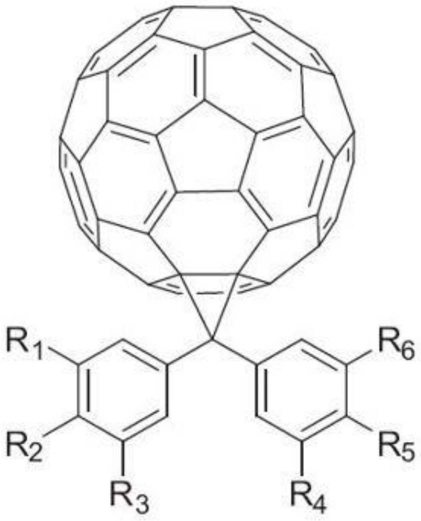

27*	H	$COOCH_3$	H	H	OC_8H_{17}	OC_8H_{17}	1.37	1.38
28	H	$COOCH_3$	H	OC_8H_{17}	OC_8H_{17}	OC_8H_{17}	1.5	1.20
29	H	$COOCH_3$	H	H	OC_8H_{17}	H	1.05	0.36
30	H	$COOCH_3$	H	H	OCH_3	OCH_3	0.22	-0.10
31	$COOCH_3$	H	$COOCH_3$	H	OC_8H_{17}	OC_8H_{17}	1	1.20
32*	H	CN	H	H	OC_8H_{17}	OC_8H_{17}	0.84	1.45
33	H	NO_2	H	H	OC_8H_{17}	OC_8H_{17}	0.51	0.86
34*	H	SO_2CH_3	H	H	OC_8H_{17}	OC_8H_{17}	1.24	1.32
35	H	SO_2CF_3	H	H	OC_8H_{17}	OC_8H_{17}	0.86	0.16

<table>
<tr><td colspan="9">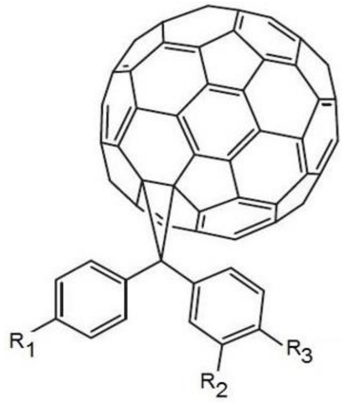
</td></tr>
<tr><td>36</td><td>$COOCH_3$</td><td>OC_8H_{17}</td><td>OC_8H_{17}</td><td>-</td><td>-</td><td>-</td><td>1.6</td><td>1.06</td></tr>
<tr><td colspan="9">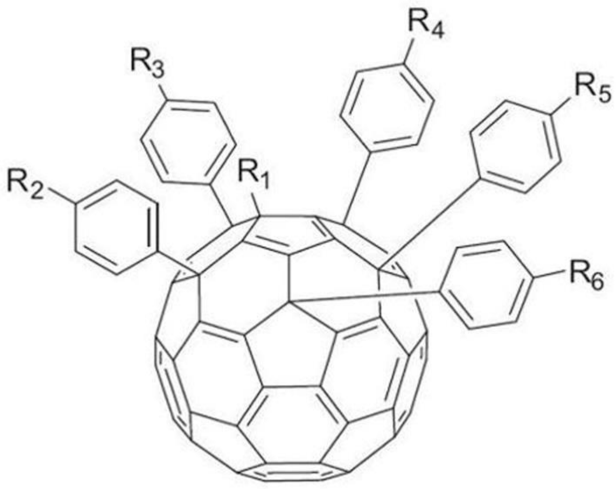
</td></tr>
</table>

37	H	C_6H_5	C_6H_5	C_6H_5	C_6H_5	C_6H_5	0.02	0.30
38	CH_3	C_6H_5	C_6H_5	C_6H_5	C_6H_5	C_6H_5	0.45	0.30
39*	CH_3	$-OC_6H_5$	$-OC_6H_5$	$-OC_6H_5$	$-OC_6H_5$	$-OC_6H_5$	1.08	1.17
40	CH_3	C_4H_9	C_4H_9	C_4H_9	C_4H_9	C_4H_9	0.91	0.10
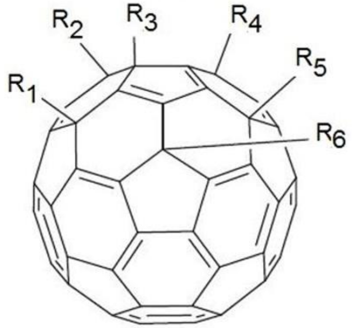								
41	CH_3	CH_3	C_8H_{17}	CH_3	CH_3	CH_3	0.15	0.82

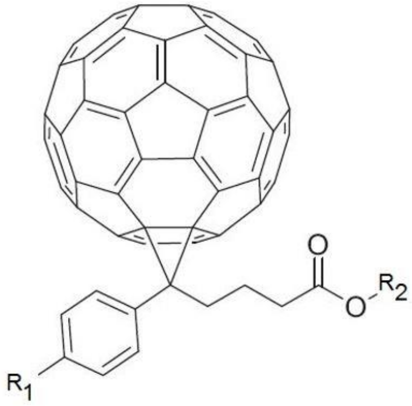									
42*	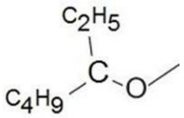	H	-	-	-	-	1.73	1.71
43	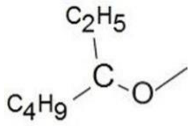	CH_3	-	-	-	-	1.01	1.83
44	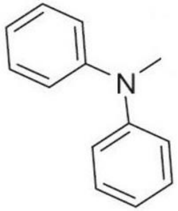	CH_3	-	-	-	-	4	2.92

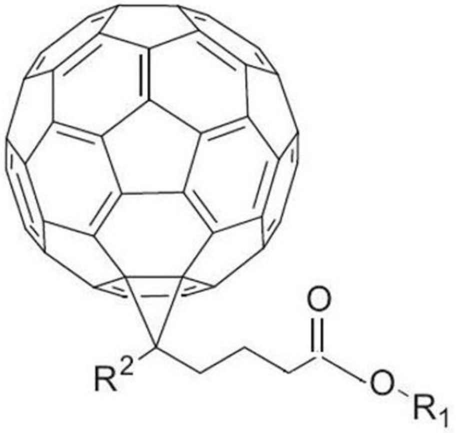									
45	C_4H_9	C_6H_5	-	-	-	-	2.45	2.71
46	C_8H_{17}	C_6H_5	-	-	-	-	1.27	1.76
47*	$C_{12}H_{25}$	C_6H_5	-	-	-	-	1.04	0.86
48*	$C_{16}H_{33}$	C_6H_5	-	-	-	-	0.11	-0.09
49	CH_3	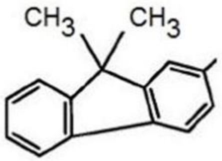	-	-	-	-	4	4.16

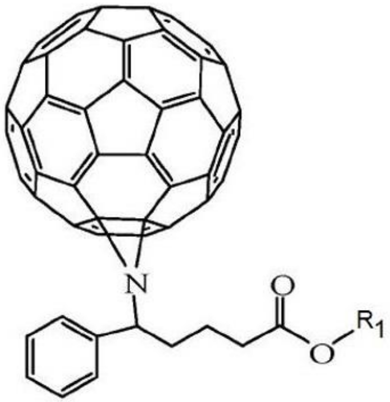								
50	CH_3	-	-	-	-	-	2.3	1.95
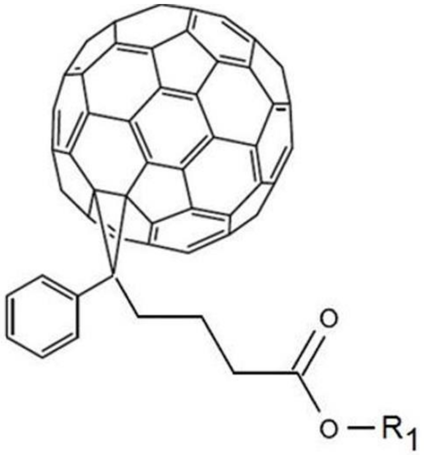								

51*	CH_3	-	-	-	-	-	4.1	3.01
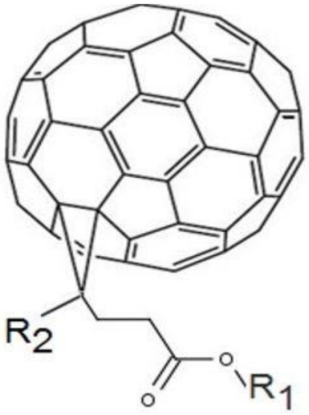								
52	CH_3	C_6H_5	-	-	-	-	1.2	1.52
53	C_2H_5	C_6H_5	-	-	-	-	1.7	1.61
54	C_4H_9	C_6H_5	-	-	-	-	1.7	1.35
55	C_3H_7	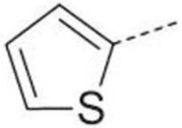	-	-	-	-	1.1	2.35
56*	C_4H_9	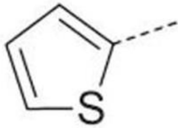	-	-	-	-	1.1	2.15

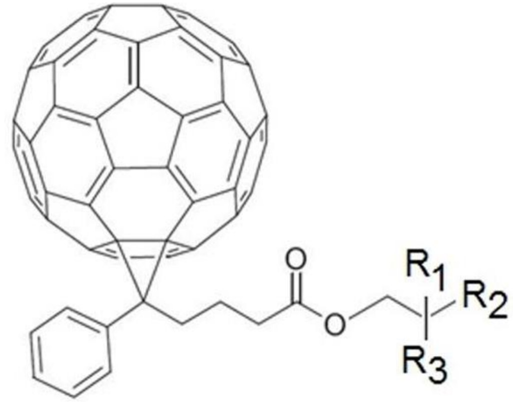								
57	CH_3	CH_3	CH_3	-	-	-	2.74	3.10
58	CH_3	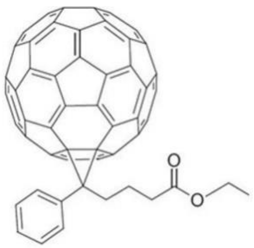	CH_3	-	-	-	3.7	3.05
59	CH_3		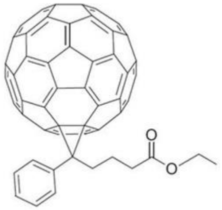	-	-	-	2.46	2.54

		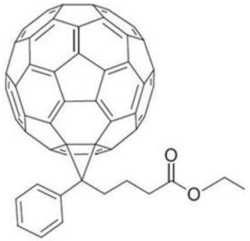						

materials

MDPI

Table S2. Computed value of modeled descriptors for each FDs along with their predicted % PCE and mean normalized distance value.

ID	D1	D2	D3	D4	D5	D6	D7	PCE (%)	FD Type	Mean Normalized Distance
FD1	12	0	0	6	0	0	286	16.62	C_{60}	0.995
FD2	12	0	0	6	0	0	203	17.28	C_{60}	0.638
FD3	6	0	8	2	0	8	137	14.96	C_{60}	0.316
FD4	12	0	8	4	0	8	274	23.01	C_{60}	0.988
FD5	8	0	0	4	0	0	167	12.10	C_{60}	0.462
FD6	8	0	0	4	0	0	176	12.03	C_{60}	0.504
FD7	8	0	0	4	0	0	166	12.11	C_{70}	0.46
FD8	8	0	0	4	0	0	229	11.61	C_{70}	0.765
FD9	4	0	0	1	0	0	119	7.96	C_{70}	0.239
FD10	8	0	0	2	0	0	244	13.36	C_{60}	0.842

D1: S_A(chg)/A_D_D_D/1_2s,1_3s,3_4a/6
D2: Fr5(chg)/B_C_C_C_D/1_4s,2_3s,2_4s,3_4s/
D3: Fr5(type)/C.3_C.3_C.3_C.3_H/1_2s,2_3s,3_4s,4_5s/
D4: Fr5(att)/C_C_E_E_E/1_3s,2_4s,3_5a,4_5a/
D5: Fr5(type)/C.3_C.3_C.AR_C.AR_C.AR/1_4s,2_3s,2_5s,4_5a/
D6: S_A(type)/C.3_C.3_C.3_C.AR/1_3s,2_3s,3_4s/5
D7: ASA_P
